# High levels of nitric oxide and peroxynitrite with protein nitration: a new therapeutic target for gliomas

**DOI:** 10.4103/mgr.MEDGASRES-D-25-00365

**Published:** 2026-09-12

**Authors:** Ying Yang, Qi Zhang, Yuchen Ma, Serge Yannick Ouedraogo, Liang Chen, Xianquan Zhan

**Affiliations:** 1 Shandong Provincial Key Laboratory of Precision Oncology, Shandong Cancer Hospital and Institute, Shandong First Medical University & Shandong Academy of Medical Sciences, Jinan, Shandong Province, China; 2 Shandong Provincial Key Medical and Health Laboratory of Ovarian Cancer Multiomics, & Jinan Key Laboratory of Cancer Multiomics, Shandong First Medical University & Shandong Academy of Medical Sciences, Jinan, Shandong Province, China; 3 Laboratory of Cancer Proteoformics, School of Pharmaceutical Sciences, Shandong First Medical University & Shandong Academy of Medical Sciences, Jinan, Shandong Province, China; 4 Department of Gynecological Oncology, Shandong Cancer Hospital and institute, Shandong First Medical University & Shandong Academy of Medical Sciences, Jinan, Shandong Province, China

**Keywords:** Cx43, gap junctions, glioma, nitroproteomics, NO, personalized medicine, protein nitration, proteoformics, signaling pathway, therapeutic drug, therapeutic target

## Abstract

The gap junction signaling pathway is a core hub mediating intercellular communication, which plays a vital role in maintaining tissue homeostasis, and its dysfunction is closely related to glioma. Nitroproteomics studies have shown that protein tyrosine nitration plays an important role in the progression of glioma, and gap junction is one of the significantly enriched nitroprotein signaling pathways in glioma; proteoformics reveals that nitration can produce various proteoforms of related proteins, which have different functional states in different subtypes of glioma, suggesting that nitration may alter the functional states of related proteins in gap junction pathways. When levels of nitric oxide and peroxynitrite (ONOO^–^) rise, they induce the tyrosine nitration of key proteins in the gap junction pathway; this nitration not only interferes with the function of the gap junction pathway, but also affects other gap junction-linked signaling networks. Forming an attack network on the gap junction pathway through multi-pathway cross-regulation has great potential for therapeutic strategies targeting the gap junction pathway. We recommend in-depth studies of the gap junction signaling pathway and potential drugs targeting gap junction signals, including drugs that target gap junction communication activators, nitration inhibitors, and drugs that directly target specific nitroproteins, combined with conventional chemotherapy or immune checkpoint inhibitors, as well as the construction of personalized medical frameworks as important future research directions.

## Introduction

Glioma is a type of primary intracranial tumor originating from neuroglial cells, accounting for 40% to 50% of intracranial tumors.^1^ The World Health Organization classification of gliomas is grades I to IV. Of them, grades I and II are low-grade gliomas, which have lower malignant potential; whereas grades III and IV are high-grade gliomas, with glioblastoma multiforme being the most malignant and invasive.^2^ Currently, most treatment options primarily include surgical resection, followed by postoperative chemoradiotherapy combined with temozolomide (TMZ). Although it is effective, the therapeutic efficacy still faces many challenges.^3^ For example, the heterogeneity of gliomas results in significant differences in treatment outcomes among different subgroups; the blood–brain barrier hinders most therapeutic drugs from reaching effective concentrations, and glioma cells are highly resistant to TMZ, among other issues.^4^,^5^,^6^ Therefore, in-depth exploration of glioma pathogenesis and the discovery of new drug targets are urgent.

Multiomics technologies provide new perspectives for investigating glioma mechanisms and identifying novel targets. Currently, a series of omics analyses have been conducted for gliomas, including genomics (identifying the mutations of isocitrate dehydrogenase 1/2,^7^,^8^ promoter mutations of telomerase reverse transcriptase, and promoter methylation status of O^6^-methylguanine-DNA methyltransferase (MGMT)),^6^,^9^ transcriptomics (RNA sequencing),^10^,^11^,^12^ epigenomics (whole-genome methylation sequencing),^13^ proteomics (protein expression profiles and phosphorylation signaling networks),^14^,^15^ nitroproteomics,^16^,^17^ metabolomics,^15^,^18^,^19^ proteoformics,^20^,^21^,^22^,^23^ etc. These integrated analyses have constructed molecular networks for gliomas, revealing alterations in some signaling pathway networks involved in glioma pathogenesis, including epigenetics, signaling pathways, oxidative stress, and metabolic reprogramming.

One post-translational modification closely related to nitrative stress has become a research focus in recent years,^16^ which mainly involves the protein tyrosine nitration mediated by reactive nitrogen species (RNS). When gliomas undergo nitrative stress, a large amount of highly reactive peroxynitrite (ONOO^–^) is produced, which attacks the phenolic ring of protein tyrosine residues, causing electrophilic addition and introducing a nitro group (-NO_2_), thereby generating 3-nitrotyrosine in a protein. This nitration can significantly alter the chemical properties and functions of target proteins, leading to various malignant effects such as disrupting the cytoskeleton, inducing drug resistance, and interfering with signaling pathways.^17^,^24^ The nitroproteomics analysis revealed that gap junction is one of the signaling pathways with significant enrichment of nitroproteins,^16^ which is composed of connexins forming cell communication channels. Among them, connexin 43 (Cx43) is the main subtype in the central nervous system and plays a central role in maintaining tissue homeostasis.^25^,^26^ In gliomas, gap junction function exhibits a complex double-edged sword: functional gap junctions have tumor-suppressing roles, whereas their dysfunction or nitration of Cx43 may promote the malignant progression of gliomas.^16^,^17^ These studies provide new possibilities for discovering drug targets and developing novel treatment strategies for gliomas. This review summarizes the gap junction signaling pathway network involved in glioma pathogenesis and its implications for discovering new drug targets and treatment approaches.

## Search Strategy

A literature search was conducted to identify studies addressing nitric oxide (NO), ONOO^−^, protein nitration, and their association with gap junction signaling pathways in glioma. The search was primarily performed in the PubMed database, with a time range from January 2010 to December 2024 to capture recent advances. Key terms were combined using Boolean operators: “glioma” or “glioblastoma” with “nitric oxide,” “peroxynitrite,” “protein nitration,” “3-nitrotyrosine,” “gap junction,” “connexin 43,” “Cx43,” “signaling pathway,” “inducible nitric oxide synthase (iNOS),” “nitroproteomics,” and “proteoformics.” For therapeutic strategies, additional terms including “therapeutic target,” “drug resistance,” “chemotherapy,” “radiotherapy,” and “blood–brain barrier” were incorporated for cross-referencing. Initial screening was based on title and abstract evaluation to assess relevance to the core topics of nitrative stress, gap junction pathways, glioma pathogenesis, and treatment modalities. Relevant literature identified in the initial screening was subjected to full-text evaluation to confirm the presence of detailed descriptions concerning specific molecular mechanisms, signaling networks, or therapeutic strategies. Inclusion criteria comprised original research or high-quality review articles published in English that directly addressed nitrative stress, gap junction dysfunction, or related targeting strategies in glioma. Exclusion criteria included non-English publications, studies not focused on glioma, those lacking explicit reference to protein nitration or core gap junction pathways, and non-peer-reviewed materials such as conference abstracts or short communications. To ensure temporal relevance and representativeness, over 85% of the cited references were published after 2018.

## Association between Glioma Subtypes and Protein Tyrosine Nitration

Protein tyrosine nitration levels were increased in parallel with glioma World Health Organization grade, a correlation driven by dysregulation of the NO/ONOO^−^ axis.^17^,^27^ Exacerbation of nitrative stress in high-grade gliomas reflected an imbalance in NO and ONOO^−^ generation, consumption, and interconversion dynamics.^27^

### Nitric oxide metabolism and peroxynitrite formation as key determinants of nitration levels

ONOO^−^ constitutes the principal chemical effector of protein tyrosine nitration.^28^ In high-grade gliomas, upregulated iNOS expression drove elevated NO production,^29^,^30^ while mitochondrial dysfunction and NADPH oxidase activation in the tumor microenvironment concurrently increased superoxide (O_2_·^−^) availability.^31^ Rapid reaction between NO and O_2_·^−^ promoted the ONOO^−^ formation, providing the immediate chemical basis for nitrative modifications.^28^ Consequently, elevated nitration observed in high-grade tumors represented a direct consequence of augmented ONOO^−^ generation.^32^ Extensive ONOO^−^ formation resulted in marked NO consumption, accounting for situations in which free NO steady-state concentrations did not scale linearly with iNOS expression but were instead channeled into formation of nitrating ONOO^−^.^28^,^33^ Furthermore, ONOO^−^ instability led to decomposition into nitrite and nitrate, explaining concomitant nitrite accumulation^28^,^34^; however, nitrite accumulation was a downstream outcome of ONOO^−^ formation and decay rather than a primary driver of nitration.^35^

### Nitrative modifications as feedback regulators of nitric oxide/peroxynitrite axis

Nitrative stress exerted critical feedback on the NO/ONOO^−^ metabolic axis.^28^ ONOO^−^-mediated nitration of NO synthases, including iNOS, induced enzyme activity attenuation, establishing a negative feedback loop.^28^ This mechanism refined NO production flux and limited unchecked accumulation. In addition, within hypoxic tumor regions characteristic of high-grade gliomas, nitrite derived from ONOO^−^ decomposition was subject to enzymatic reduction by multiple reductases, resulting in NO re-release.^36^ Such a local cycle of ONOO^−^ formation is capable of replenishing NO to sustain pro-survival and pro-angiogenic signaling^37^ (**Figure 1**).

**Figure 1:**
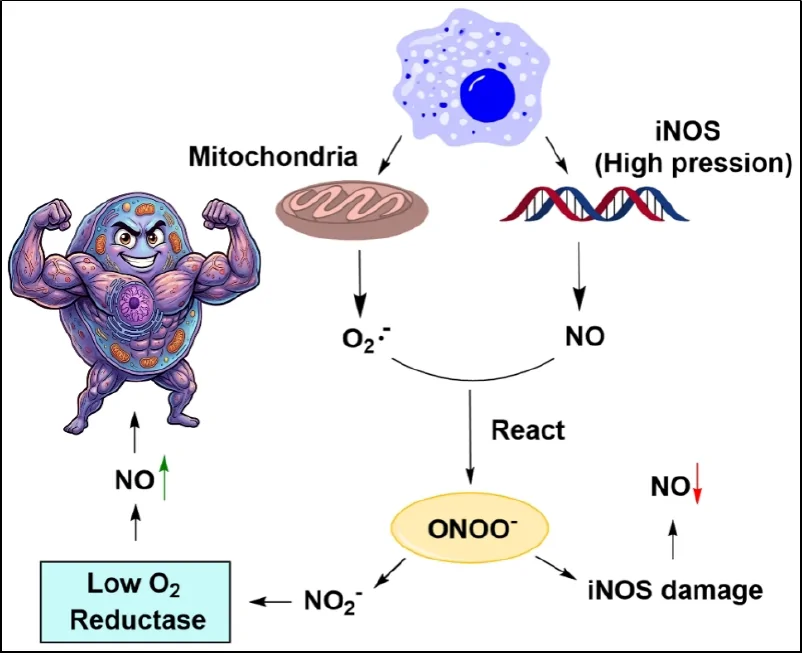
NO–ONOO^−^ metabolic axis imbalance in glioma contributes to protein tyrosine nitration and establishment of feedback regulatory mechanisms. iNOS: Inducible nitric oxide synthase; NO: nitric oxide; NO_2_^–^: nitrite; O_2_.^–^: superoxide anion; O_2_: dioxygen; ONOO^−^: peroxynitrite. Created with Microsoft Office Powerpoint 2019 and ChemDraw.

## Molecular Composition and Nitration with Nitrogen-Containing Reactive Gases of Gap Junction Pathway

### Molecular composition of gap junction pathway

The gap junction signaling pathway can mediate direct intercellular communication and maintain tissue homeostasis.^25^,^38^ The key hub affecting this pathway is the connexin family, among which Cx43 has been widely studied. Cx43 is mainly expressed in the central nervous system, particularly with high expression in astrocytes.^39^,^40^ The peptide chain of Cx43 protein consists of an N-terminus, a C-terminus, two extracellular loops, four transmembrane domains, and one intracellular loop.^41^,^42^ The basic functional unit is the connexon, a hemichannel formed by the arrangement of six Cx43 proteins,^43^,^44^ which can dock with adjacent hemichannels to form a complete gap junction channel. This allows the direct passage of small molecules such as ions (Ca^2+^, K^+^, Na^+^, etc.),^45^,^46^ second messengers [cyclic adenosine monophosphate (cAMP), inositol trisphosphate, and cyclic uridine monophosphate],^47^ metabolites (glucose and amino acids),^48^,^49^ and microRNAs. Through direct communication via the gap junction signaling pathway, it efficiently coordinates cellular group behavior, maintains metabolic coupling and electrophysiological synchronization, significantly enhancing tissue homeostasis.^50^,^51^

### Nitration mechanism of the gap junction pathway

In the pathological microenvironment of gliomas, persistent inflammatory responses and nitrative stress lead to the excessive production of RNS, among which the abnormal accumulation of ONOO^−^ is the core driver of nitrative stress.^52^,^53^ Nitrative stress is essentially a pathological state characterized by the irreversible nitration at tyrosine residues in a protein mediated by ONOO^−^, resulting in the formation of 3-nitrotyrosine.^54^ Nitration not only alters the physicochemical properties of proteins but, more importantly, when it targets key signaling pathway proteins, it triggers a series of cascading reactions that lead to cellular dysfunction and malignant phenotypes.^55^,^56^ In gliomas, the carboxyl-terminal domain of the gap junction protein Cx43 is rich in tyrosine residues, making it a primary target for nitrative stress attacks.^57^ Studies have shown that the carboxyl-terminal domain of Cx43 protein is abundant in tyrosine residues, and ONOO^−^ can nitrate the phenolic hydroxyl groups of these tyrosine residues to form 3-nitrotyrosine.^58^,^59^ This modification significantly alters the physicochemical properties of tyrosine residues, including increasing molecular volume, altering local charge distribution, and affecting hydrogen bond formation capability. Protein tyrosine nitration is an irreversible post-translational modification that has profound effects on the conformation and function of Cx43 protein. ONOO^−^-mediated nitration modifies the conformation of Cx43 through multiple mechanisms: the introduction of the nitro group increases the molecular volume and hydrophobicity of tyrosine residues, leading to steric hindrance or allosteric effects at binding sites with proteins, such as zonula occludens-1 and Src,^54^ causing conformational rearrangement of local peptide chains; nitration changes the pKa value of the phenolic hydroxyl group of tyrosine, making it prone to deprotonation under normal pH conditions, thereby affecting the hydrogen bond network and electrostatic interactions.^54^ Nitration may expose or mask specific protein binding sites (such as proline-rich regions or PDZ-binding motifs), altering the interactions of Cx43 with other proteins.^59^,^60^ The tyrosine residues in the carboxyl-terminal domain of Cx43 play a key role in maintaining protein conformation and functional regulation. These residues are not only involved in protein–protein interactions but also affect the phosphorylation state and channel gating properties of Cx43.^61^ Nitration introduces bulky -NO_2_, which interfere with conformational changes of the Cx43 carboxyl terminus through steric hindrance effects, thereby affecting its interactions with regulatory proteins (such as kinases and phosphatases), ultimately leading to abnormal gap junction channel function.^62^ Cx43 nitration directly inhibits the assembly and function of gap junction channels. Nitrated Cx43 exhibits altered distribution on the cell membrane, forming abnormal assemblies, resulting in changes in channel gating properties, manifested as reduced channel opening probability, decreased single-channel conductance, and altered permeability.^63^ Nitration can promote the internalization of Cx43 from the membrane, entering early endosomes via clathrin-mediated endocytosis and being degraded by lysosomes, significantly reducing the number of functional Cx43 on the membrane.^64^,^65^

### Nitration-mediated regulation of non-gap functions of connexin 43

The classic function of Cx43 is to form intercellular channels for signal transmission.^66^ However, a study has found that nitrated Cx43 shifts from the cell membrane to the mitochondria and nucleus.^67^ In the mitochondria, it inhibits apoptosis by interfering with the permeability transition pore, while in the nucleus, it may influence proliferation-related genes.^67^,^68^ Additionally, nitration disrupts the interaction between Cx43 and the cytoskeletal protein zonula occludens-1, leading to cell polarity disruption and enhancing the invasiveness of glioma cells.^69^ Nitration also causes Cx43 to lose its normal regulatory function on the Hippo/yes-associated protein pathway,^70^ which can act as a scaffold protein to enhance the sustained activation of the epidermal growth factor receptor signaling pathway.^71^ These changes collectively drive the malignant progression of glioma.

## Nitration with Nitrogen-Containing Reactive Gases in Gliomas via Multi-Pathway Cross-Regulation

Nitration in gliomas exerts a three-dimensional network on the gap junction core protein Cx43 through multiple signaling pathways.^54^,^72^,^73^ Activation of mitogen-activated protein kinase/extracellular signal-regulated kinase (MAPK/ERK), phosphatidylinositol 3-kinase/protein kinase B (PI3K/AKT), protein kinase C (PKC), and cyclic adenosine monophosphate/protein kinase A (cAMP/PKA) pathways is associated with NO and ONOO^−^ levels. Direct nitration of tyrosine residues in Cx43 by ONOO^−^ induces conformational changes in the protein.^54^,^72^ This not only directly damages the assembly and function of gap junction channels but also significantly alters the sensitivity of Cx43 to interact with the intracellular kinase/phosphatase network^74^ (**Figure 2**). In the MAPK/ERK pathway, ONOO^−^ inhibits the activities of protein tyrosine phosphatases, leading to sustained activation of the ERK pathway.^74^ Activated ERK specifically phosphorylates serine residues (such as Ser255, Ser279, and Ser282) at the carboxyl terminus of Cx43, while nitration of Cx43 further enhances the sensitivity of these serine phosphorylation sites through steric hindrance and allosteric effects, forming a synergistic amplification mechanism of nitration-phosphorylation.^75^,^76^ In the PI3K/AKT pathway, ONOO^−^ nitrates the active site Cys124 in phosphatase and tensin homolog deleted on chromosome 10 (PTEN) to inhibit its activity,^77^ leading to abnormal activation of AKT. Activated AKT phosphorylates the residue Ser373 in Cx43,^78^ and the conformational change of Cx43 modified by nitration makes this site more susceptible to recognition and phosphorylation by AKT, accelerating the ubiquitination degradation of Cx43.^78^ Additionally, ONOO^−^ can also enhance PKC kinase activities through nitration.^79^,^80^,^81^ Activated PKC directly phosphorylates the residue Ser368 in Cx43,^79^,^81^ and the conformational change of Cx43 induced by nitration exposes this site, making it more susceptible to PKC attack.^79^,^80^ Meanwhile, ONOO^−^ nitrates the active center of adenylate cyclase, inhibiting cAMP production,^82^ weakening the positive regulation of PKA on Cx43 function,^83^ and the conformational change of nitrated Cx43 also reduces its response to PKA-mediated stabilizing phosphorylation.^83^ Nitration with other modifications, such as ubiquitination, acetylation, and SUMOylation, occurs in complex dynamic ways.^66^ For example, nitration may influence the acetylation or SUMOylation of nearby lysine residues through steric hindrance,^66^,^84^ while pre-existing acetylation could alter tyrosine’s sensitivity to nitration.^84^ Nitration may also serve as a degradation signal, making Cx43 more recognizable by ubiquitin ligases and thus accelerating its degradation via the proteasome.^85^ Currently, it is unclear whether nitration cooperates, antagonizes, or cascades with other modifications, and whether this ultimately promotes tumor suppression or malignant progression. Future research should employ quantitative proteomics and gene editing to systematically map the modification landscape of Cx43, clarifying the driving role of nitration within the multi-modification network and providing new avenues to develop multi-target regulatory drugs.

**Figure 2:**
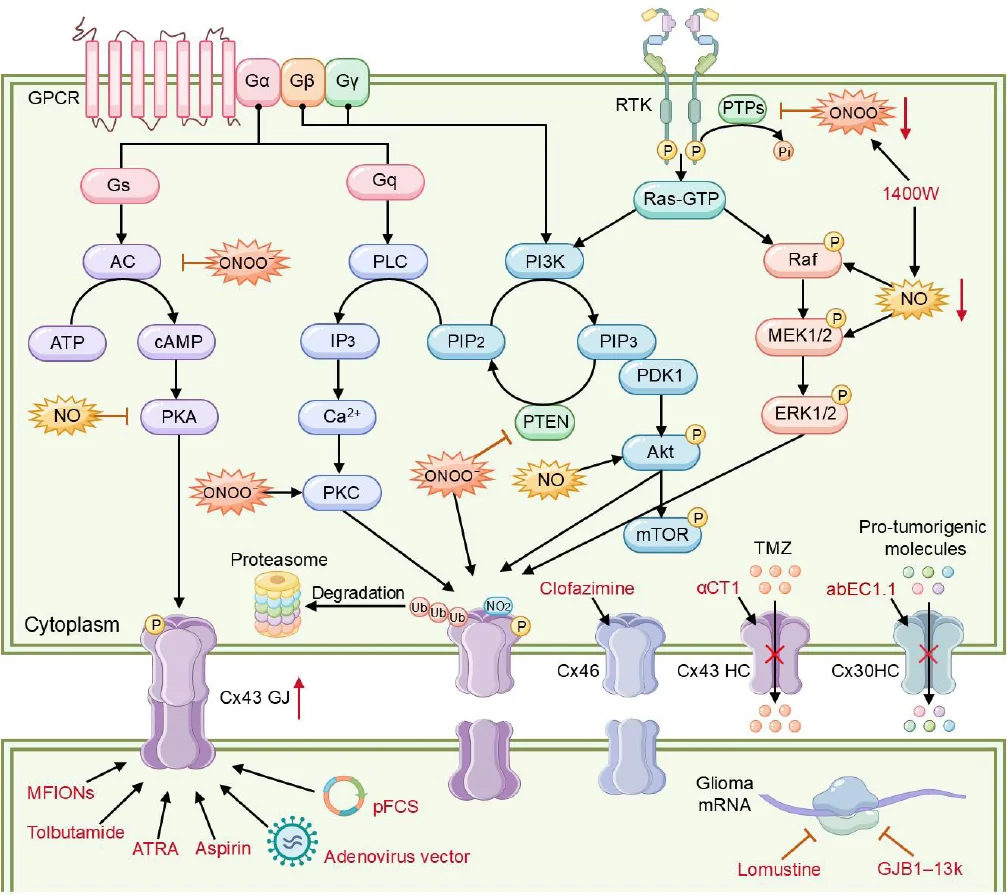
Cx43-mediated GJ communication and multipath cross-regulatory network. AC: Adenylate cyclase; AKT: protein kinase B; ATRA: all-trans retinoic acid; cAMP: cyclic adenosine monophosphate; Cx30HC: connexin 30 hemichannel; Cx43: connexin 43; Cx43HC: connexin 43 hemichannel; Cx46: connexin 46; Cys124: cysteine 124; ERK: extracellular signal-regulated kinase; GJ: gap junction; GJB1-13k: subtype of Cx32; GPCR: g protein-coupled receptor; GTP: guanosine triphosphate; IP3: inositol trisphosphate; MAPK: mitogen-activated protein kinase; MEK1/2: mitogen-activated protein kinase kinase 1/2; MFIONs: magnetic fluorescent iron oxide nanoparticles; mTOR: mechanistic target of rapamycin; NO: nitric oxide; ONOO^−^: peroxynitrite; P: phosphate; PDK1: 3-phosphoinositide-dependent protein kinase 1; pFCS: phosphorylated focal adhesion kinase; PI3K: phosphatidylinositol 3-kinase; PIP2: phosphatidylinositol 4,5-bisphosphate; PIP3: phosphatidylinositol (3,4,5)-trisphosphate; PKA: protein kinase A; PKC: protein kinase C; PLC: phospholipase C; PTEN: phosphatase and tensin homolog deleted on chromosome 10; PTPs: protein tyrosine phosphatases; RTK: receptor tyrosine kinase; Ser: serine; TMZ: temozolomide; Ub: ubiquitin. Created with Microsoft Office PowerPoint 2019.

## Multigroup Classification of Gap Junction Pathway in Gliomas

Based on the functional status of the gap junction pathway and integrated multiomics data, gliomas can be divided into three subtypes: C1 (high nitration-functionally inactive), C2 (hemichannel hyperactive-metabolically dysregulated), and C3 (functionally stable).^86^,^87^,^88^ Among them, the C1 subtype is the most distinct but has a poorer prognosis.^59^,^62^ Nitroproteomics analysis confirmed high expression of iNOS in this glioma subtype, leading to excessive production of NO and its derivative ONOO^−^, which specifically attack and nitrosylate the tyrosine residues of Cx43.^60^,^86^ This post-translational modification directly disrupts the stability and structural assembly of the Cx43 protein. Meanwhile, transcriptomic and phosphoproteomic data revealed abnormal hyperactivation of the MAPK/ERK and PI3K/AKT/mammalian target of rapamycin (mTOR) signaling pathways in this subtype.^63^,^88^ Activated ERK and AKT kinases further phosphorylate serine sites of Cx43, and this aberrant phosphorylation synergizes with nitrosylation to inhibit Cx43’s ability to form full channels, accelerating its ubiquitination degradation,64 thereby rendering the bystander effect mediated by gap junctions ineffective.^65^ Apoptotic signals (such as toxic metabolites produced by TMZ chemotherapy) are thus unable to spread between glioma cells via the gap junction pathway, allowing a large number of bystander glioma cells to survive.^65^,^89^ This is reflected in the shortest progression-free survival and overall survival in C1 patients.^10^,^90^ The C2 subtype is characterized by abnormal opening and hyperactivity of Cx43 hemichannels. Transcriptomic and metabolomic analyses showed a significant increase in gene sets related to hemichannel function in this subtype, leading to substantial leakage of signaling molecules such as adenosine triphosphate and glutamate into the glioma microenvironment.^91^,^92^ These signaling molecules can activate P2X purinergic receptors 7 and N-methyl-D-aspartate receptors in the microenvironment, thereby driving downstream calcium signaling, pro-inflammatory signaling, and pro-invasive signaling pathways.^93^,^94^ Metabolic reprogramming promotes glycolysis to produce large amounts of lactate, causing severe acidification of the microenvironment, which greatly inhibits gap junction channel function and provides an optimal microenvironment for glioma cell proliferation. Therefore, C2 gliomas are typically highly invasive.^95^,^96^,^97^ The C3 glioma subtype has low nitration levels, and protective signaling pathways such as cAMP/PKA remain active.^98^ PKA phosphorylation of serine sites at the C-terminal of Cx43 helps stabilize the open conformation of gap junction channels and promotes their binding to tight junction protein-1, thereby maintaining relatively intact intercellular communication function.^99^ This cellular population synergy enables effective diffusion of apoptotic signals, increasing glioma cell apoptosis, so patients with this subtype respond better to conventional chemotherapy and radiotherapy and have relatively better clinical outcomes^100^ (**Figure 3**).

**Figure 3:**
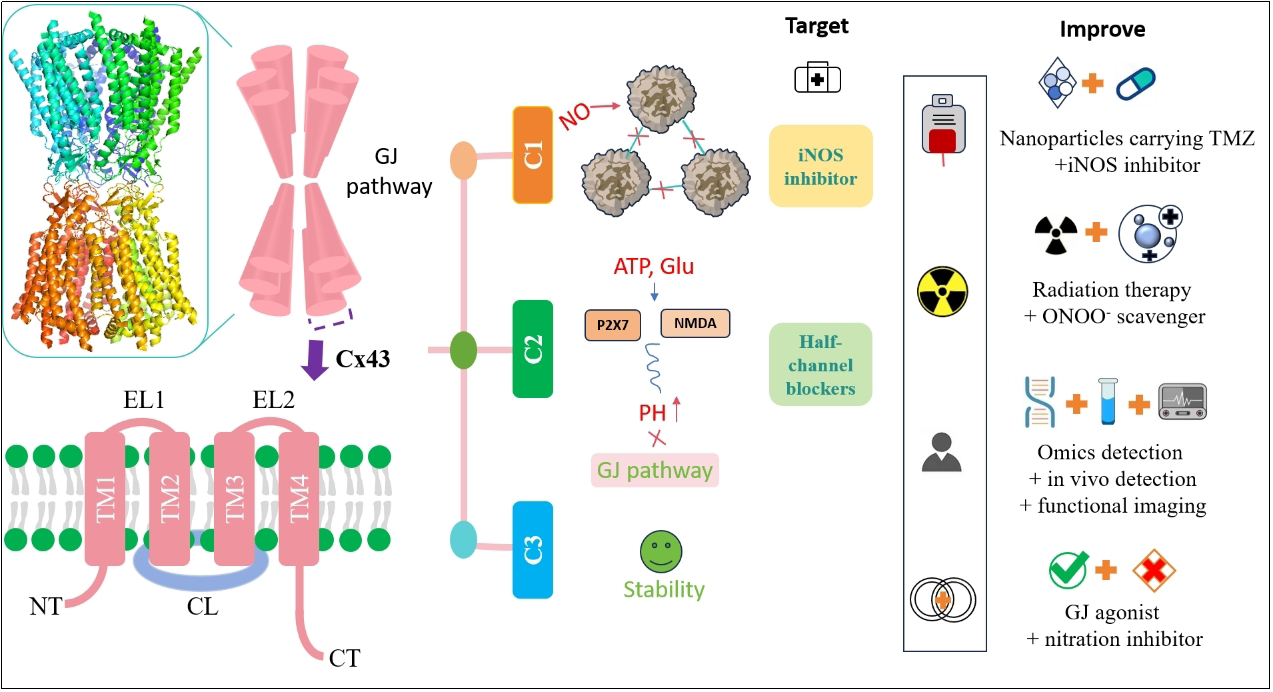
Molecular architecture of Cx43, functional classification of gliomas based on the GJ pathway, and corresponding therapeutic targeting strategies. ATP: Adenosine triphosphate; CL: cytosolic loop; Cx43: connexin 43; EL1/2: extracellular loop 1/2; GJ: gap junction; Glu: glutamate; iNOS: inducible nitric oxide synthase; NMDA: N-methyl-D-aspartate receptor; NO: nitric oxide; NT: N-terminus; ONOO^−^: peroxynitrite; P2X7: P2X purinoceptor 7; PH: potential of hydrogen; TM1–TM4: transmembrane helix 1–4; TMZ: temozolomide. Created with Microsoft Office PowerPoint 2019.

## Potential Therapeutic Targets in Gap Junction Pathway for Glioma Treatment

The signaling network regulatory factors and their components of the gap junction signaling pathway are all potential targets for glioma treatment.

(i) iNOS is the source of nitration. In the glioma microenvironment, the activation of iNOS catalyzes the production of large amounts of NO and ONOO^−^,^56^ which directly attack the tyrosine residues of Cx43, disrupting the structure and stability of the gap junction pathway,^87^ leading to pathway dysregulation. This further inhibits the bystander effect mediated by the gap junction pathway, preventing surrounding glioma cells not directly attacked by drugs from receiving apoptosis signals, significantly reducing the mortality of glioma cells.^65^,^101^

(ii) AKT is a key component of PI3K/AKT/mTOR pathway. When the tumor suppressor protein PTEN is inhibited, phosphatidylinositol 3,4,5-trisphosphate increases, recruiting and activating AKT.^88^ Activated AKT phosphorylates the serine sites of Cx43 protein in gap junction pathway, causing conformational changes in the protein that inhibit the formation of its full channels. Moreover, phosphorylated Cx43 protein is recognized and degraded by ubiquitin ligases, significantly reducing the amount of Cx43,^102^ completely interrupting gap junction signaling pathway and preventing intercellular communication, thereby slowing the apoptosis rate of glioma cells.^100^

(iii) Abnormally open Cx43 hemichannels are a key factor affecting C2 gliomas. Abnormally open Cx43 hemichannels lead to the leakage of large amounts of signaling molecules such as adenosine triphosphate and glutamate.^90^,^92^ Excessive adenosine triphosphate activates P2X purinergic receptors 7 receptors, causing calcium influx and driving pro-inflammatory and pro-invasive signals,^103^ while excessive glutamate activates N-methyl-D-aspartate receptors, leading to neuronal hyperexcitability and epilepsy.^94^ At the same time, abnormally open Cx43 hemichannels leak some metabolites, causing glioma cells to produce large amounts of lactate, exacerbating the acidification of the glioma cell microenvironment,^96^ thereby inhibiting the function of Cx43 full channels and making glioma cells even more difficult to eliminate.^95^

(iv) Programmed death protein-1 (PD-1) is an immune checkpoint protein. When its ligand programmed death-ligand 1 (PD-L1) binds to it, T cells receive a stop signal, T cell function stops, proliferation is hindered, and cytokine secretion decreases,^104^ thereby preventing glioma cells from being recognized and killed, significantly reducing the mortality of glioma cells.^105^,^106^

A multi-level therapeutic framework targeting gap junction pathway damage caused by nitration is formed. Based on the above regulatory mechanisms of nitration on gap junction signaling pathway, the treatment of gap junction pathway damage caused by nitration will mainly focus on four aspects: (i) targeting the upstream source of nitration; (ii) regulation of gap junction pathway protein expressions; (iii) regulation of downstream channel function; and (iv) optimization of traditional therapies.^39^ Nitration originates from the expression of iNOS, which serves as the upstream source target. The regulatory proteins of the gap junction pathway can be used as direct drug targets, and the regulatory factors of other downstream signaling pathway networks can also serve as targets for targeted therapy (**Additional Table 1**).^107^,^108^,^109^,^110^,^111^,^112^,^113^,^114^,^115^,^116^,^117^,^118^,^119^,^120^,^121^,^122^,^123^,^124^,^125^,^126^,^127^,^128^,^129^,^130^,^131^,^132^,^133^ The overall drug treatment strategy is to regulate gap junction channel activity and protect normal communication while blocking pro-cancer signals.^134^ Optimization of traditional therapies involves continuous innovation based on existing radiotherapy and chemotherapy techniques, aiming for precise treatment.

**Additional Table 1:** Pharmacological agents for ameliorating nitration-mediated GJ impairment

Targeted strategy category	Drug	Molecular mechanism	Biological effect	Research model	Reference
Suppressing the source of nitration	1400W	iNOS inhibitor	Directly inhibits glioma stem cell growth and induces autophagy	U87 cells	107
	Geranylgeranylacetone	Upregulate TRX mRNA and protein levels, downregulate TXNIP	Inhibits NLRP3 inflammasome activation through denitrosylation	GBM cells, mice	108
Regulation of Cx43 expression	5-Azacitidine	A cytidine analogue that covalently binds and degrades methyltransferases	When used in combination with TMZ, it exhibits synergistic antitumor effects	IDH1 R132H mutant cells; PDX model	109
	Decitabine (5-aza-2-deoxycytidine)	A cytosine analogue covalently binds and degrades methyltransferases	Induction of apoptosis through exogenous, endogenous, and JAK/STAT pathways	GBM cells	110
	Lomustine	Add alkyl groups to DNA and RNA to interfere with transcription and translation	When used in combination with TMZ, it demonstrates synergistic therapeutic effects and improves survival rates	MGMT promoter methylation in glioblastoma patients	111
	Adenovirus vector	Insert GJA1 cDNA into the vector to upregulate Cx43 expression.	Suppressing the malignant phenotype of GSCs	Glioma stem cells, mice	112
	pFCS plasmid vector	Cx43 expression vector, upregulates Cx43 expression	Cell proliferation rate slows down, tumorigenicity decreases	GBM cells, mice	113
	Quercetin	Hsp27 Inhibitor	Inhibiting Hsp27 sensitizes glioblastoma to TMZ	GBM cells	114
	Targeted Nanogel	Cx43 binds to specific antibodies, providing anchorage sites for nanogel	Promote tumor site enrichment to enhance therapeutic efficacy	GBM cells, rats	115
Targeting other connexins	Clofazimine	Specific inhibition of Cx46-mediated GJA1 conductance	Inhibit tumor growth and reduce tumor volume	Mice	116
	GJB1–13k	A subtype of Cx32 that inhibits overall mRNA translation	Inhibit tumor proliferation and promote apoptosis	GBM cells, mice	117
	Cx30	Block glucose metabolic reprogramming in gliomas	Reduce the energy supply to tumors and inhibit proliferation	Rats, glioma patient samples	118
Channel adjustment function	Tolbutamide	Inhibit KATP channels, upregulate Cx43, enhance GJA1 ion conductance GJIC	Enhancing the bystander effect of HSV-tk/GCV therapy	U373 cells	119
	Magnetosome-like ferrimagnetic iron oxide nanochains	Enhance HSV-tk expression and Cx43 levels to strengthen GJIC	Enhance the bystander effect of HSV-tk/GCV therapy	C6 cells, rats	120
	Aspirin	Reduce prostaglandin E2 synthesis, upregulate Cx43, enhance GJIC	Reduce the invasiveness of gliomas	C6 cells	121
	All-trans retinoic acid	Enhance GJIC functionality	Significantly enhance the efficiency of intercellular communication	C6 cells	122
	abEC1.1	Inhibition of Cx26, Cx30, and Cx32 hemichannels	Inhibit tumor proliferation and invasion, reduce the release of tumor-promoting molecules	Mice, patient GBM cells	123
	αCT1	Inhibits Cx43 hemichannels and promotes Cx43 aggregate formation	When used in combination with TMZ, it enhances its sensitivity	3D hydrogel GBM organoid model	124
Combination therapy strategy	Tf-PO-DOX	Tf can cross the blood-brain barrier, and PO is an ideal drug carrier	The drug efficiently crosses the blood-brain barrier, enhancing delivery efficiency	C6 cells, rats	125
	MnO_2_-DOX@mT/OMVs	OMVs can cross the blood-brain barrier, MnO_2_ induces ferroptosis	Integrate chemotherapy with chemokinetics to achieve synergistic antitumor effects	C6 cells, rats	126
	MTX@EVs-KLA-LDL	KLA peptide is an apoptosis-inducing therapeutic peptide, while MTX is a chemotherapy drug	Integrate chemotherapy with peptide therapy for synergistic antitumor effects	U87 cells, mice	127
	Blue light-irradiation *in situ* gelation hydrogel platform	Integrated chemotherapy, photothermal therapy, and radiotherapy	Through the synergistic effects of multiple therapies, GBM is significantly suppressed	U87-MGFL cells, MBR-614 cells, mice	128
	Cela@Lip-IRH	IR780 targets mitochondria and mediates photodynamic therapy, while Cela induces apoptosis	Combined chemotherapy and photodynamic therapy	C6 cells, mice	129
	CM-M1	Downregulation of MGMT and MPG expression	When used in combination with TMZ, it exhibits synergistic therapeutic effects	A172 and T98G GBM cell lines	130
	^18^F-DOPA PET-guided MRI-directed short-course high-dose-rate proton beam therapy	Integrates precision imaging guidance with advanced radiotherapy technology	Significantly extend patient survival while maintaining quality of life and cognitive function.	Newly diagnosed GBM patients aged 65 years and older	131
	Closo-ortho-carborane-based glucosamine derivative	Carborane as a boron carrier exhibits high boron content.	Boron enrichment efficiency surpasses the clinical gold standard boronophenylalanine	U87 cells	132
	Stereotactic radiosurgery	Deliver ionizing radiation precisely to the smallest therapeutic volume	Its use after recurrence may confer survival benefits	Patients with high-grade glioma	133

^18^F-DOPA: Fluorine-18 fluorodopa; 3D: three-dimensional; cDNA: complementary DNA; Cela@Lip-IRH: celastrol-loaded lipid nanoparticles; Cx: connexin; DOX: doxorubicin; GBM: glioblastoma; GCV: ganciclovir; GJ: gap junction; GJA1: gap junction protein alpha 1; GJB1-13k: subtype of Cx32; GJIC: gap junction intercellular communication; GSCs: glioma stem cells; Hsp27: heat shock protein 27; HSV-tk: herpes simplex virus thymidine kinase; IDH1: isocitrate dehydrogenase 1; iNOS: inducible nitric oxide synthase; JAK: Janus kinase; KATP: ATP-sensitive potassium channel; MGMT: O6-methylguanine-DNA methyltransferase; MnO_2_: manganese dioxide; MPG: N-methylpurine DNA glycosylase; MRI: magnetic resonance imaging; MTX@EVs-KLA-LDL: methotrexate-loaded extracellular vesicles engineered with both a therapeutic peptide and a targeting peptide; NLRP3: NOD-, LRR- and pyrin domain-containing protein 3; OMVs: outer membrane vesicles; PDX: patient-derived xenograft; PET: positron emission tomography; pFCS: phosphorylated focal adhesion kinase; PGE2: prostaglandin E2; PO: poly(ortho ester); STAT: signal transducer and activator of transcription; Tf: transferrin; TMZ: temozolomide; TXNIP: thioredoxin-interacting protein.

### Treatment strategies to inhibit the source of nitration

Overexpression of iNOS is the starting point of nitration. Targeting iNOS prevents the excessive generation of NO and ONOO-, thereby curbing the interference of nitration on gap junction pathways at the source.^56^ Selective iNOS inhibitors can bind to the active site of iNOS with high affinity, which competitively inhibits the conversion of the substrate L-arginine, reduces the concentration of NO,^135^ decreases the generation of ONOO-, alleviates nitration damage to the Cx43 protein of gap junction pathways,86 blocks the abnormal activation of downstream signaling pathway kinases, and further protects the stability and functional integrity of the CX43 protein.^136^ Denitrase agonists can also significantly reduce the impact of nitration. Their mechanism involves specifically activating endogenous denitrase to directly remove nitro groups, thereby diminishing damage to the Cx43 protein.^137^,^138^ This strategy is particularly suitable for the treatment of C1 gliomas, greatly reducing the subsequent damage caused by nitration.^139^

### Therapeutic strategies to target connexin 43 in gap junction pathway

In glioma treatment, Cx43 is the most severely disrupted protein by nitration. It can promote intercellular transmission of chemotherapy drug toxicity signals by forming functional channels, as well as regulate the cell cycle and inhibit angiogenesis, thereby suppressing tumor progression in multiple dimensions.^140^ Currently, intervention strategies targeting Cx43 mainly focus on three technical approaches: epigenetic modifiers (e.g., DNA methyltransferase inhibitors), gene therapy (e.g., virus vector-mediated Cx43 overexpression), and small-molecule drugs (e.g., quercetin).^141^,^142^

(i) Epigenetic modification achieves precise control of Cx43 expression by regulating the promoter activity of gap junction protein alpha 1 (*GJA1*) gene, serving as an important strategy for glioma treatment. Hypermethylation of GJA1 promoter is a key factor leading to downregulation of Cx43 expression and maintenance of malignant cell phenotype.^112^ DNA methyltransferase inhibitors, such as decitabine and 5-azacytidine, can irreversibly inhibit DNA methyltransferase activity or incorporate into DNA strands to competitively prevent methylation, reversing promoter hypermethylation, and thus restoring Cx43 expression and gap junction function,^109^,^143^,^144^ alleviating the damage and inhibition of Cx43 protein by nitration. In isocitrate dehydrogenase 1-mutant gliomas, the combination of 5-azacytidine and TMZ can significantly enhance chemosensitivity and inhibit tumor proliferation.^109^,^145^ Lomustine, by interfering with transcription and translation processes, shows synergistic efficacy with TMZ in glioblastoma patients with MGMT promoter methylation.^111^

(ii) Gene therapy employs viral or non-viral vectors to overexpress Cx43 in glioma cells, reconstructing gap junction pathway.^146^ In the application of viral vectors, the thymidine kinase (*TK*) gene from the herpes simplex virus/ganciclovir system mediates the diffusion of toxic metabolite GCV-TP through gap junction intercellular communication (GJIC) while also facilitating apoptotic body phagocytosis.^147^ Overexpressed Cx43 can reconstruct a functional gap junction pathway, thereby counteracting downstream signaling abnormalities (e.g., ERK/AKT) induced by nitration and reducing the degradation of Cx43 protein.

(iii) Small-molecule drugs, with their favorable safety and ease of use, can effectively target Cx43. Quercetin can enhance the sensitivity of TMZ by inhibiting Hsp27, and their combined use can significantly induce apoptosis in glioma cells.^114^ The TMZ/quercetin nanoemulsion exhibits excellent drug release and penetration properties in *in vitro* studies, effectively reducing the half-maximal inhibitory concentration (IC_50_) value.^148^ In addition, cisplatin nano-gel, through synergistic action with Cx43 and the brain-specific anion transporter (BSAT1), significantly inhibits glioma growth.^115^ Clinical drugs such as dexamethasone and levetiracetam can also exert auxiliary antitumor effects by interfering with Cx43-mediated cell communication.^149^

### Therapeutic strategies to target other proteins in gap junction pathway

Other proteins in gap junction pathway also provide new strategies for glioma treatment. The Cx46 protein is highly concentrated in the glioma stem cells,^150^ which are the root cause of tumor recurrence and drug resistance. Reducing Cx46 significantly impairs the self-renewal ability and *in vivo* tumor-forming capacity of glioma stem cells. Designing a Cx46-targeting peptide based on the extracellular domain of Cx46 offers a promising new strategy for glioma treatment.^116^,^151^ A subtype of the Cx32 connexin protein can inhibit glioblastoma by suppressing overall mRNA translation,^117^,^152^ while the anesthetic propofol protects tumor cells from cisplatin-induced killing by inhibiting the Cx32 protein and can also inhibit Cx43 and Cx26 proteins, affecting the function of the gap junction signaling pathway.^153^ Therefore, protective agents related to CX32 protein can serve as therapeutic agents for glioma, while also paying attention to the side effects of certain anesthetics during combination therapy. Cx30 can also affect cellular glucose metabolism and mediate metabolic reprogramming to influence disease progression,^118^ but once Cx30 undergoes radiotherapy, it helps gliomas resist treatment.^154^ This also provides direction for glioma treatment. In addition, Clofazimine can specifically inhibit Cx46-mediated gap junction communication, thereby suppressing tumor growth.^154^ The subtype of Cx32 (GJB1-13k) can inhibit global mRNA translation or block the glucose metabolism reprogramming of tumor cells by interfering with Cx30 function, both of which provide new ideas for the precise treatment of glioma.^117^,^118^

### Treatment strategies to functionally regulate gap junction downstream pathways

The regulation of the gap junction downstream pathways mainly occurs through two forms: GJIC and cell surface hemichannels.^155^ Therefore, regulatory strategies targeting the gap junction downstream pathway in gliomas primarily focus on activating functional GJIC and inhibiting pathological hemichannels.

Activating functional GJIC is an important strategy to enhance the therapeutic effect on glioma.^156^ Interventions such as all-trans retinoic acid, iron oxide nanoparticles, and aspirin can effectively upregulate Cx43 expression, enhance protective signaling pathways like cAMP/PKA that are inhibited by nitration stress,^119^,^157^,^158^ stabilize the open conformation of Cx43, and promote the formation of GJIC, thereby significantly increasing the phototoxic effect of PDT and enhancing the “bystander effect” of toxic metabolites in suicide gene systems (such as TK gene from herpes simplex virus/ganciclovir).^119^,^120^,^121^,^122^,^158^

Inhibiting the excessive opening of pathological Cx43 hemichannels is a key step to prevent the malignant progression of gliomas. Nitration and its activated PKC pathway are critical for the abnormal opening of Cx43 hemichannels. Using specific inhibitors (such as abEC1.1) to block the opening of pathological Cx43 hemichannels can effectively prevent ATP/glutamate leakage without affecting the function of full channels, thereby effectively inhibiting the progression of glioma.^123^ TMZ is a common drug for treating gliomas, but it has certain resistance. Combining abEC1.1 to inhibit Cx43 hemichannels may reduce the efflux of TMZ and increase the sensitivity of gliomas, thereby improving the treatment method.^123^ Regulating glioma gap junction channels requires a bidirectional strategy: first, activating GJIC through genetic intervention or drugs to restore normal intercellular communication and inhibitory signal transmission; second, using drugs or molecular targeting to inhibit hemichannels to block their mediated excitotoxicity and pro-tumor effects.

## Targeted Nitrogen-Containing Reactive Gases for Nitration-Mediated Gap Junction Pathway in the Innovation and Optimization of Glioma Treatments

### Nitration and chemotherapy in glioma

The chemotherapy for gliomas is primarily based on TMZ, but its efficacy is often limited by the blood-brain barrier, tumor heterogeneity, and drug resistance.^159^ To overcome these limitations, chemotherapy strategies are shifting from traditional cytotoxic drug applications to co-delivery systems that involve precise drug delivery systems and active modulation of tumor microenvironment. The introduction of nanotechnology provides a systematic solution:

(i) Intelligent delivery systems achieve targeted drug delivery through functional design. For example, transferrin receptor-targeted nanoparticles can enhance the enrichment of doxorubicin at the tumor site^125^; natural carriers such as exosomes, with their biocompatibility, offer optimized solutions for brain delivery.^160^ Postoperative local drug delivery strategies, such as gelatin hydrogels, can achieve sustained drug release and maintain effective concentrations.^128^

(ii) Combined treatment strategies enhance efficacy through multi-mechanism synergy. Near-infrared laser-activated nanoparticles can simultaneously achieve chemotherapy and photothermal therapy, directly damaging the mitochondria of tumor cells.^129^ Personalized medication targeting molecular phenotypes such as low expression of MGMT and N-methylpurine-DNA glycosylase can significantly enhance the DNA damage efficiency of TMZ.^130^ The most promising approach is targeting the nitrative stress microenvironment. For C1 gliomas, a co-delivery nano-platform can simultaneously load TMZ and nitrative stress inhibitors (such as iNOS inhibitors). This strategy not only directly kills tumor cells but also reduces the nitration of Cx43 by inhibiting ONOO^−^ generation, restoring the function of the gap junction pathway.

### Nitation and radiotherapy in glioma

Radiotherapy is a core adjuvant modality after glioma surgery, but its application still faces the dual challenges of tumor radioresistance and normal tissue injury.^161^,^162^ Technological advancements have driven radiotherapy toward precision and personalization: proton therapy can significantly reduce damage to normal brain tissue^163^; boron neutron capture therapy enhances treatment precision through biological targeting^164^,^165^; closo-carborane-based glucosamine derivatives, whose boron enrichment efficiency in tumor cells is also excellent^132^; new models such as preoperative stereotactic radiosurgery demonstrate potential in controlling tumors while preserving neurological function.^166^ The interaction between radiotherapy and tumor microenvironment provides a new perspective for overcoming resistance. Radiotherapy may induce bursts of RNS, which alter the function of connexins (such as Cx30, Cx43) through nitration, thereby weakening efficacy. Therefore, radiotherapy combining nitrative stress inhibitors (such as iNOS inhibitors) may reverse this process, restore gap junction function, and enhance radiotherapy sensitivity. For C1, radiotherapy can be combined with nitrative stress inhibitors. For C2, hemichannel blockade is consideredfor combination with radiotherapy. For C3, nitrative stress inhibitors can maximize the inherent radiotherapy sensitivity. In addition, magnetosome-like ferrous oxide magnetic nanoparticle chains and tolbutamide enhance the bystander effect of suicide gene therapy by upregulating Cx43 and enhancing GJIC.^119^,^120^ The Cela@Lip-IRH (celastrol-loaded lipid nanoparticles) nanoplatform in combination with chemotherapy with photodynamic therapy,^129^ and MTX@EVs-KLA-LDL (methotrexate-loaded extracellular vesicles engineered with both a therapeutic peptide and a targeting peptide) integrating chemotherapy with pro-apoptotic peptide therapy,^127^ demonstrate synergistic antitumor effects in preclinical glioma models.

### Nitration and other combined treatments in glioma

To achieve precise and effective targeting of gliomas, a personalized medical framework based on a combined treatment strategy is proposed. The core of this framework lies in designing synergistic treatment regimens and dynamically applying them according to individual patient characteristics. Synergistic treatment design is an inevitable path toward clinical application. First, in terms of synergy with standardized chemotherapy/radiotherapy, the combination of gap junction agonists with TMZ can overcome chemotherapy resistance by enhancing the bystander effect.^67^ Blue light-irradiated in-situ gel hydrogel platform can achieve the integration of multimodal therapies at the tumor site.^128^ CM-M1 can downregulate DNA repair enzymes (MGMT and N-methylpurine-DNA glycosylase).^130^ They exhibit synergistic therapeutic effects when used in combination with TMZ. Nitration is a significant root cause of TMZ resistance.^11^ Gap junction agonists in combination with NO synthase inhibitors (such as iNOS-selective inhibitors) restore intercellular communication and reduce nitration damage to Cx43 protein,^86^ producing a synergistic sensitization effect, which also applies to combination with radiotherapy. Second, in terms of synergy with immunotherapy, modulating gap junctions can influence the tumor immune microenvironment,^167^ while nitrative stress is a major driver of glioma immune evasion.^168^ Therefore, jointly targeting gap junctions and nitrative stress (such as ONOO^−^ scavengers) can more effectively reverse immunosuppressive states like T-cell dysfunction, thereby generating strong synergy with immune checkpoint inhibitors like PD-1/PD-L1 and reshaping the immunosuppressive microenvironment.^169^ Finally, in multi-pathway synergistic targeting, the nitration signaling axis can be regarded as a core synergistic target. For example, designing a combined regimen that simultaneously inhibits Src kinase (regulating Cx43 phosphorylation) and iNOS (an upstream source of nitration) may achieve more comprehensive and three-dimensional regulation of Cx43 function.

### Synergy and antagonism in combination therapeutic regimens

Combined targeting of nitrative stress with conventional therapies in glioma is associated with both synergistic and antagonistic outcomes.^170^ Synergy with radiotherapy and chemotherapy is primarily mediated by inhibition of iNOS or scavenging of ONOO^−^, resulting in attenuation of Cx43 nitrative modification and restoration of gap junction–mediated bystander effect, thereby facilitating propagation of cytotoxicity induced by TMZ or ionizing radiation.^171^,^172^ A potential adverse consequence is stabilization of blood-brain barrier integrity secondary to iNOS inhibition, with resultant impairment of chemotherapeutic delivery.^173^,^174^ In combination with immunotherapy, suppression of nitrative stress enables reversal of T-cell exhaustion and potentiation of immune checkpoint inhibitor efficacy.^175^,^176^ However, inappropriate iNOS inhibition may compromise macrophage-mediated functions and engender antagonistic effects.^171^ Multi-target strategies directed at the nitration signaling axis (such as combination of iNOS and Src inhibition) permit more comprehensive modulation of Cx43 function and prevention of tumor escape via signaling re-wiring,^123^,^177^ but entail increased risk of off-target toxicity and pharmacokinetic interactions.^178^ Overall, fine optimization of combination regimens is required to maximize synergistic effects while minimizing antagonistic interactions.

### Assessment of pharmacokinetic risks and neuroimmune effects

iNOS inhibitors and modulators of nitrative stress for glioma are predominantly at the preclinical stage, and brain pharmacokinetic properties remain poorly characterized. Systematic evaluation of blood–brain barrier penetration, differential distribution and metabolism in tumor versus normal brain tissue, and potential pharmacokinetic/pharmacodynamic interactions with chemotherapeutics such as TMZ is required. Neurotoxicity constitutes a central safety concern because NO functions as a neurotransmitter and regulator of cerebrovascular tone; nonselective iNOS inhibition risks impairment of neurovascular homeostasis.^171^ Development of highly selective iNOS inhibitors is therefore imperative. Potential long-term effects of iNOS inhibition on neural stem cell compartments and higher cognitive function warrant incorporation of neurocognitive monitoring into clinical protocols.^171^ Given the dichotomous roles of NO in immune regulation, inhibition of iNOS may suppress tumor-associated nitrative stress while concurrently compromising macrophage and T cell effector functions.^179^,^180^ Therapeutic regimens thus require precise optimization of dosing and scheduling to balance antitumor efficacy with preservation of host defense, thereby maximizing therapeutic benefit.

### Potential influence of glioma heterogeneity and disease progression on targeted nitrative therapy

The nitrative state and functional subtypes of gliomas may undergo dynamic remodeling, representing a key mechanism underlying acquired resistance to nitrative targeting therapies.^181^ Nitrative status is dynamically regulated by the tumor microenvironment, particularly inflammation, hypoxia, and oncogenic signaling.^182^ Application of nitrative targeting (such as iNOS inhibition) imposes selective pressure that drives adaptive reprogramming in tumor cells.^183^,^184^ For instance, tumors initially classified as high-nitrative, functionally inactive (C1 subtype) may reduce nitrative burden through downregulation of iNOS and upregulation of antioxidant defenses,^185^,^186^ or activate parallel pathways (such as PKC) to sustain gap junction dysfunction,^187^ thereby transitioning into low-nitrative but persistently malignant C2- or C3-like subtypes that evade therapeutic intervention. This phenotypic plasticity contributes to resistance through three mechanistic layers: (1) Target loss: Reduction in intratumoral nitrative load diminishes susceptibility to iNOS/ONOO^−^-directed agents.^188^ (2) Signal rewiring: Alternative post-translational modifications (such as phosphorylation) substitute for nitration to maintain inactivation of critical proteins (such as Cx43), preserving oncogenic signaling output.^123^,^189^ (3) Clonal selection: Therapeutic pressure enriches for clones independent of nitrosative stress, such as those exhibiting pathological hemichannel opening (a C2 feature).^190^,^191^

### Nitration and personalized medical framework in glioma

A personalized medical application is key to achieving precise targeting. Its successful implementation relies on two pillars: predictive biomarkers and dynamic monitoring systems. In terms of predictive biomarkers, it is essential to establish a precise patient stratification model that goes beyond traditional Cx43 expression and phosphorylation status. This requires integrating the specific nitration levels of Cx43, features in systemic circulation (such as sorcin and other specific nitroproteins), and biomarkers of its nitration load (such as 3-nitrotyrosine levels^54^) to construct a biomarker panel based on multiomics (especially nitroproteomics^192^), thereby more accurately predicting patient sensitivity to gap junction function enhancement, hemichannel inhibitors, or nitration-targeted therapies.^88^ The analysis of connexin nitration patterns in patient serum or tissue samples using proteoformics can serve as a biomarker to more accurately reflect the functional status of the gap junction pathway.^20^,^21^,^22^,^23^ In terms of dynamic monitoring and adaptive treatment, real-time monitoring of treatment response and resistance emergence is achieved through functional imaging (such as specific positron emission tomography probes) or liquid biopsy techniques.^193^ If elevated tumor nitrative stress levels are detected during treatment, it indicates the need to promptly add or switch to nitrative stress inhibitors, thereby enabling dynamic adjustment of the treatment regimen to ensure sustained therapeutic effectiveness.

## Protein Nitration and Clinical Research on Glioma

We searched clinical trials targeting protein nitration, NO/ONOO^−^ stress, and gap junction signaling for glioma treatment at ClinicalTrials.gov (https://clinicaltrials.gov/) and the Chinese Clinical Trial Registry (ChiCTR; https://www.chictr.org.cn/index.html) with keywords including glioma, glioblastoma, nitric oxide, peroxynitrite, protein nitration, nitrotyrosine, Cx43, gap junction, iNOS inhibitors, and targeted therapy.

Current registered trials show that clinical translation of protein nitrationtargeted therapy for gliomas remains at a very early stage. As of the search date, no registered clinical trials specifically investigating iNOS inhibitors, ONOO^−^ scavengers, denitrase agonists, or gap junction/Cx43 modulators have been identified in either platform. Moreover, although iNOS inhibitors show therapeutic potential in preclinical glioma models, no clinical trial data have been published to date.

Current advances in this field are mainly concentrated in preclinical studies and basic mechanistic exploration. Most candidate drugs have been tested in preclinical models in combination with TMZ, radiotherapy, or immune checkpoint inhibitors to reverse chemo and radioresistance by alleviating nitrative stress and restoring GJIC. A study has confirmed that the iNOS/NO axis is critical for maintaining stemness of glioma stem cells and mediating TMZ resistance; targeted inhibition of iNOS can effectively sensitize gliomas to TMZ and eliminate resistant cells.^194^ In addition, the STING agonist 8803 significantly prolongs survival in preclinical glioblastoma models by reprogramming the tumor immune microenvironment.^195^

Overall, clinical trials directly targeting specific protein nitration sites or nitrated proteins remain scarce, indicating a noticeable gap between basic nitroproteomics and protein morphomics research and clinical practice. In fact, protein tyrosine nitration is a selective and posttranslational modification associated with oxidative stress. Nitroproteomics based on gel electrophoresis and tandem mass spectrometry can effectively identify nitrated proteins and biomarkers in astrocytomas, and has been used to elucidate the functional characteristics of nitrated proteins, nitrationmediated signaling networks, and tumor diagnosis and treatment, thereby providing a theoretical basis for targeting protein nitration in glioma therapy.

Major current challenges include the lack of highly selective nitrationtargeted drugs, the absence of a standardized patient stratification system, and the lack of unified methods for *in vivo* detection of nitration levels. Future clinical trial design should integrate nitroproteomics and proteoformics to develop subtypestratified precision therapeutic strategies, and systematically validate the clinical value of protein nitration as a predictive biomarker and therapeutic target for gliomas.

## Limitations

Molecular mechanistic characterization remains at a conceptual level, with dynamic definition insufficient. A macro-scale portrait of nitration-mediated formation of a multidimensional stereoscopic attack network via multi-pathway cross-regulation is presented; however, precise molecular evidence delineating dynamic crosstalk between nitration and phosphorylation or other posttranslational modifications is lacking.

Clinical translational pathway for gap junction-based molecular subtyping remains indistinct. The proposed C1–C3 functional subtypes provide mechanistic insight but are predominantly inferred from data-driven analyses and lack clinical validation. Subtype-specific nitration sites and functional status of Cx43 and other gap junction components have not been precisely mapped.

Therapeutic strategies confront practical translational barriers. Candidate agents, including highly selective iNOS inhibitors and hemichannel blockers, and proposed combination regimens are subject to dual bottlenecks. First, brain-targeted delivery efficiency, intratumoral accumulation, and blood–brain barrier perturbation have not been sufficiently evaluated in advanced preclinical models. Second, complex synergistic and antagonistic interactions between nitration inhibitors and conventional radiochemotherapy or emerging immunotherapies, together with long-term modulation of neuroimmune microenvironment, remain highly uncertain.

Biomarker identification and clinical validation are insufficient. Although a precisionmedicine paradigm integrating multiomics and liquid biopsy is proposed, translation of nitroproteomics and other complex molecular data into clinically deployable, robust biomarker panels for routine assays and realtime therapeutic stratification currently lacks feasible technical pathways and supporting clinical validation.

## Future Perspectives

Future research on glioma needs to advance multidimensionally in the mechanisms of protein nitration mediating gap junction pathways, treatment optimization, technological breakthroughs, and clinical translation.

High-resolution nitroproteomics and single-cell multiomics should be leveraged to delineate dynamic cross-regulatory networks of nitration, acetylation, phosphorylation, and other post-translational modifications in key proteins such as Cx43, thereby providing molecular evidence for the definition of functional subtypes C1–C3. Noninvasive liquid biopsy approaches should be developed to enable dynamic, real-time monitoring of tumor nitration status, gap junction pathway functionality and therapeutic response (**Figure 4**).

**Figure 4:**
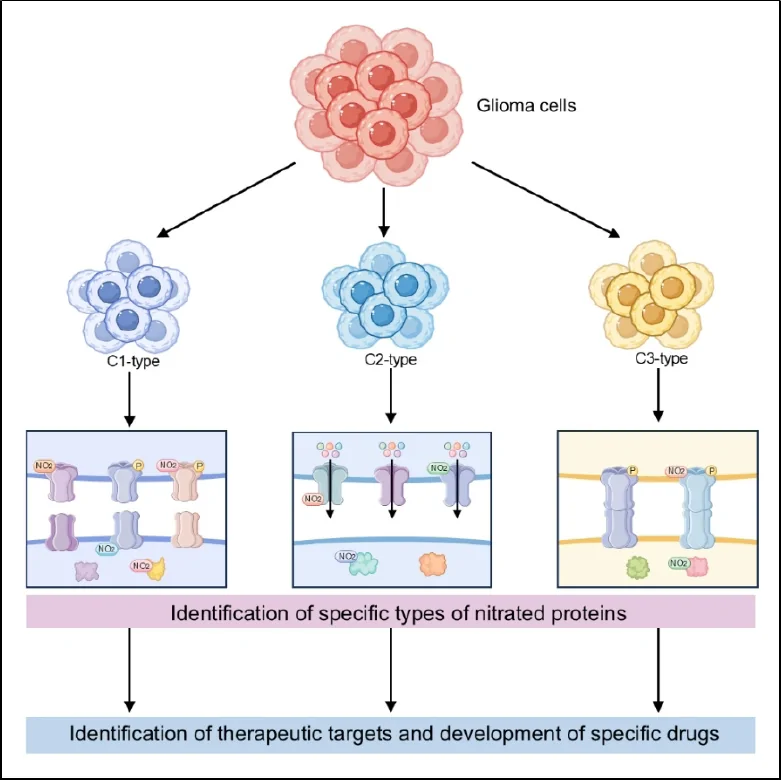
Glioma cell subtype differentiation and development pipeline for nitrated-protein-targeted therapeutics. Created with Microsoft Office PowerPoint 2019.

Development of highly selective iNOS inhibitors and brain-penetrant hemichannel modulators is required, alongside systematic screening of natural-product libraries (including traditional medicine-derived compounds) for agents with anti-nitrative stress potential to expand the therapeutic repertoire. Delivery-technology development should prioritize blood-brain barrier penetration by means of liposomes, micelles, nanoparticles and related platforms, and optimization of administration routes (sublingual, intravenous, local sustained-release) to achieve precise brain-targeted delivery and effective tumor-site accumulation.

Prospective, large-cohort clinical trials are required for systematic validation and optimization of proposed therapeutic strategies. Synergistic effects, potential antagonism and neurotoxicity associated with combined administration of nitration-targeted agents and existing modalities (radiotherapy, chemotherapy, and immunotherapy) should be evaluated, and long-term effects on neuroimmune microenvironmental composition and function should be defined to enable safe, individualized combination regimens. Integration of nitrative-stress–related biomarkers (specific nitrated proteins and metabolites) into routine clinical testing should be advanced to construct an initial diagnostic and therapeutic workflow of molecular subtyping, efficacy prediction, and resistance monitoring.

The above advances should be integrated into a complete translational closed loop spanning basic discovery to clinical application. Dynamic subtyping and monitoring should guide patient enrollment and therapeutic selection (precision decision), novel agents and delivery technologies should enable intervention (precision intervention), and clinical trial outcomes together with biomarker feedback should drive continual optimization (precision optimization), achieving deep integration of mechanistic elucidation, dynamic stratification, targeted intervention, clinical validation, and iterative optimization (**Figure 5**).

**Figure 5:**
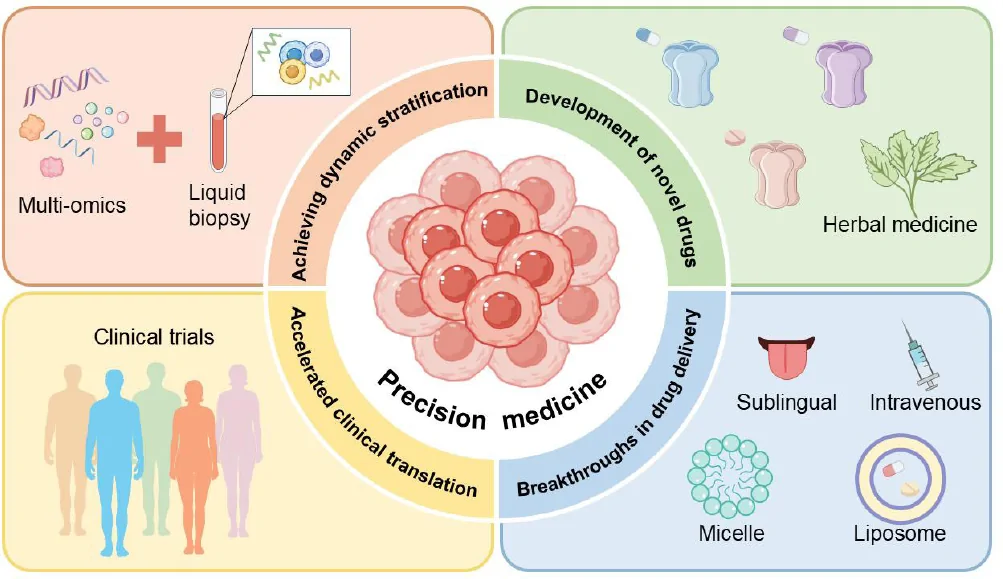
Precision medicine for future diagnostic and therapeutic strategies in glioma. Created with Microsoft Office PowerPoint 2019.

## Conclusion

Gliomas remain one of the most formidable challenges in the field of neuro-oncology due to their high heterogeneity, invasiveness, and treatment resistance. The efficacy of traditional treatment regimens primarily based on surgery and TMZ chemotherapy has reached a bottleneck, necessitating the exploration of breakthroughs at the molecular mechanism level. This review systematically elucidates how the critical pathological process of nitration profoundly influences the malignant progression and therapeutic response of gliomas by disrupting gap junction signaling pathways; and reveals that the core effector molecule (ONOO^−^) of nitration induces tyrosine nitration of connexin Cx43 and synergizes with aberrant phosphorylations of oncogenic signaling pathways such as MAPK, PI3K/AKT, and PKC, collectively compromising the stability and function of the gap junction pathway. This disruption directly leads to the failure of the crucial “bystander effect,” making it difficult for the cytotoxic effects of chemotherapy and radiotherapy to effectively spread among tumor cells, thereby exacerbating treatment resistance. The proposed functional classification of the gap junction pathway in gliomas (C1–C3 types) not only provides a new perspective for understanding tumor heterogeneity but also more importantly lays the theoretical foundation for personalized medicine. Future studies can combine proteoformics to validate and refine these subtypes. Targeted strategies should be distinctly different for various subtypes (e.g., high nitration in C1 type or hemichannel hyperactivity in C2 type). Therapeutic strategies targeting gap junction pathway damage caused by nitration demonstrate broad prospects for multi-level, combined interventions. These include upstream use of iNOS inhibitors or denitrase agonists to curb nitration at its source; pathway-level precise regulation of Cx43 expression and function through epigenetic drugs, gene therapy, or small-molecule drugs (such as quercetin); downstream bidirectional modulation of GJIC and pathological hemichannels; and finally, the integration of the aforementioned targeted strategies with conventional chemoradiotherapy and immunotherapy (such as immune checkpoint inhibitors) through advanced nano-co-delivery systems, which is expected to achieve synergistic enhancement.

## Additional file:

***Additional Table 1:*** *Pharmacological agents for ameliorating nitration-mediated GJ impairment.*

**Facts**

1. A nitrative stress–gap junction pathogenic axis is proposed.
2. A function-oriented molecular classification of gliomas is proposed based on multiomics data.
3. This process involves the cross-regulation of multiple pathways for a multidimensional target protein.
4. A multi-level targeting strategy spanning from etiological origins to clinical application has been delineated.
5. A personalized therapeutic framework for precision medicine is established.

**Open Questions**

1. Molecular mechanistic characterization remains at a conceptual level, with a dynamic definition that is insufficient.
2. Clinical translational pathway for gap junction-based molecular subtyping remains indistinct.
3. Biomarker identification and clinical validation are insufficient.

## Data Availability

*All data generated or analyzed in this study are included in this published article and its additional file.*
